# Postoperative Systemic Inflammatory Response, Complication Severity, and Survival Following Surgery for Colorectal Cancer

**DOI:** 10.1245/s10434-016-5204-5

**Published:** 2016-03-25

**Authors:** Stephen T. McSorley, David G. Watt, Paul G. Horgan, Donald C. McMillan

**Affiliations:** Academic Unit of Surgery, School of Medicine, University of Glasgow, Glasgow Royal Infirmary, Glasgow, UK

## Abstract

**Background:**

This study examined the relationship between the magnitude of the postoperative systemic inflammatory response (SIR), the severity of complications, and long-term outcomes following surgery for colorectal cancer.

**Methods:**

Data were recorded prospectively for patients undergoing potentially curative surgery for colorectal cancer in a single centre between 2008 and 2013. The magnitude of the SIR was measured using C-reactive protein (CRP). Complications were classified by Clavien-Dindo grade. The impact on disease specific and overall survival was assessed using univariate and multivariate Cox regression.

**Results:**

Of 377 patients included, the majority were male (55 %), older than age 65 years (68 %), with colonic (63 %) and node-negative disease (66 %). A total of 138 patients (37 %) had a complication, of which 26 (6 %) were Clavien-Dindo grade 3 or 4 severity. Complication severity was significantly associated with the established CRP thresholds of 150 mg/L on postoperative day (POD) 3 (*p* < 0.001) and POD 4 (*p* < 0.001). Median follow-up was 42 months with disease-specific survival 86 % and overall survival 78 %. On univariate analysis, complication severity [hazard ratio (HR) 1.66, 95 % confidence interval (CI) 1.13–2.43, *p* = 0.009], and POD 4 CRP > 150 mg/L (HR 2.53, 95 % CI 1.43–4.48, *p* = 0.001) were associated with disease-specific survival. On multivariate survival analysis, POD 4 CRP > 150 mg/L (HR 2.00, 95 % CI 1.12–3.59, *p* = 0.020), but not complication severity, was significantly associated with disease-specific survival independent of TNM stage (HR 2.46, 95 % CI 1.52–4.12, *p* < 0.001).

**Conclusions:**

The magnitude of the postoperative SIR, evidenced by CRP, was significantly associated with long-term outcomes following surgery for colorectal cancer, independent of complications and stage.

Colorectal cancer is a significant burden of disease in the developed world.^1^ Surgical resection continues to form the cornerstone of management of this disease. However, surgery is not without problems in terms of postoperative morbidity and mortality.^2^ Furthermore, it has become clear that postoperative complications following surgery for colorectal cancer are not only associated with poor short-term outcomes but also with poorer long-term outcomes.^3^–^6^

Postoperative complications have previously been defined as “deviation from the normal postoperative course.”^7^ They have been classified by type, primarily as infective or noninfective, or by severity using the Clavien-Dindo scale.^3^,^7^–^10^ There is good evidence that the type of complication, in particular infective complications, has a significant negative impact on cancer-specific and overall survival following surgery for colorectal cancer.^11^,^12^ Fewer studies have examined the impact of complication severity on long-term outcomes, although those that have reported poorer disease-free and overall survival.^13^,^14^ Indeed, a recent meta-analysis reported that severe complications had a greater impact on long-term outcomes following surgery for colorectal cancer.^15^

The magnitude of the systemic inflammatory response, as evidenced by postoperative CRP thresholds of 190 mg/L on postoperative day (POD) 2, 170 mg/L on POD 3, and 145 mg/L on POD 4, have been reported to be associated with the development of infective complications.^16^–^18^ More recently, a comprehensive review suggested that CRP concentrations above a threshold of 150 mg/L on POD 3–5 should prompt investigation and or treatment of potential postoperative complications in colorectal surgery.^19^ Two recent studies have examined the relationship between the magnitude of the postoperative systemic inflammatory response, as measured by CRP, and the severity of complications following surgery for colorectal cancer.^20^,^21^ Moreover, two recent studies in oesophagogastric cancer have suggested that CRP concentrations in the postoperative period are significantly associated with survival independent of postoperative complications.^22^,^23^ To the authors knowledge, no study investigating the interaction between the magnitude of the postoperative systemic inflammatory response and complication severity, and their impact on long-term outcomes has been performed in colorectal cancer surgery.

Therefore, the purpose of the present study was to examine the relationship between the magnitude of the postoperative systemic inflammatory response and complication severity and to determine which, if any, had the greatest impact on long-term outcomes following surgery for colorectal cancer.

## Patients and Methods

### Patients

This observational study included patients who underwent elective, potentially curative surgery for histologically confirmed colorectal cancer in a single centre between March 2008 and May 2013. Patients with metastatic disease who underwent palliative procedures or had existing inflammatory conditions were excluded.

All patients received prophylactic antibiotics and venous thromboprophylaxis prior to the induction of anaesthesia as per hospital policy. All patients were cared for in line with a unit standardised enhanced recovery after surgery (ERAS) protocol, which included preoperative carbohydrate drinks, early mobilisation, and early enteral nutrition, with the avoidance of routine peritoneal and/or nasogastric drainage. On each POD, patients were clinically assessed and had blood samples, including serum CRP, obtained as standard until discharged. Further postoperative investigation and intervention was at the discretion of the patient’s surgical team who were not blind to postoperative blood results.

## Methods

All data were collected prospectively in a database, anonymised, and subsequently analysed. Recorded information included patient demographics, tumour site, TNM stage (TNM, AJCC), surgical approach, whether adjuvant or neoadjuvant treatment was given, whether the presentation was elective or emergency, the presence of complications, preoperative serum CRP, and albumin measurements. Data regarding the nature, severity, and management of complications was retrospectively categorised using the Clavien-Dindo scale.^7^ Any uncertainties were addressed by review of electronic and/or physical case notes. Date and cause of death were crosschecked with the Registrar General (Scotland). Death records were complete until June 30, 2015, which served as the censor date. The study was approved by the West of Scotland Research Ethics Committee, Glasgow.

Serum concentrations of CRP (mg/L) were measured using an autoanalyzer (Architect; Abbot Diagnostics, Maidenhead, UK) with a lower detectable limit of 0.2 mg/L as was serum albumin (normal range 35–50 g/L). The preoperative modified Glasgow Prognostic Score (mGPS) was calculated from preoperative serum CRP and albumin.^24^

### Statistical Analysis

Categorical data regarding patient characteristics were compared using the Chi square test and Chi square test for linear association where appropriate. Patients who underwent colonic resection were analysed as a subgroup due to significant differences in postoperative complication rates between those with colonic and rectal cancers. Those patients who died within 30 days of surgery or during the same admission (Clavien-Dindo grade 5 complications) were excluded from survival analysis. Univariate and multivariate survival data were analysed using Cox’s proportional hazards model. Variables associated with disease-specific or overall survival at a significance level of *p* < 0.1 on univariate analysis were included in multivariate modelling using backward conditional regression where a two-sided *p* value <0.05 was considered statistically significant. Disease-specific survival was defined as time from date of surgery to date of cancer-specific death. Overall survival was defined as time from date of surgery to date of death from any cause. Statistical analyses were performed using IBM SPSS version 22 for Windows (Chicago, IL).

## Results

### Patients

A total of 377 patients were included having undergone potentially curative surgery for colorectal cancer in the absence of metastatic disease. The majority were male (55 %), older than age 65 years (68 %), with colonic (63 %) and node-negative disease (66 %). In total, 110 patients (29 %) had a laparoscopic resection, and the remainder had open surgery. Amongst the 138 patients with rectal cancer, 65 (47 %) with locally advanced or margin-threatening disease had neoadjuvant treatment, of which 10 (15 %) were subsequently found to have had a pathological complete response. Of all included patients, 29 % had adjuvant treatment following surgery.

### Complications

Of 377 patients, 138 (37 %) experienced complications (Table 1). Four patients (1 %) died within 30 days of surgery or during the same admission. When classified using the Clavien-Dindo scale, 108 (30 % of all patients) were grade 1–2 (i.e., required minor intervention) and 26 (6 %) were grade 3–4 (i.e., necessitated major intervention). When patient’s demographic, pathological, and clinical characteristics were compared across complication severity (Table 2), male gender (*p* < 0.01), ASA grade (*p* < 0.05), smoking status (*p* < 0.05), and rectal cancer (*p* < 0.05) were significantly associated with Clavien-Dindo grade. There was a significant association between complication severity and the proportion of patients breaching the established CRP threshold of 150 mg/L on POD 2 (*p* = 0.004), POD 3, and POD 4 (both *p* < 0.001).

**Table 1:** Postoperative complications by severity

Complication	*n*	%
No complication	239	63
Any complication	138	37
Complication type		
Infective		
All infective complications	94	25
SSI		
Wound infection	43	11.5
Anastomotic leak	16	4
Intra-abdominal abscess	6	2
RSI		
Pneumonia	23	6
Septicaemia	2	0.5
UTI	4	1
Noninfective		
All noninfective complications	44	12
Wound		
Seroma	2	0.5
Dehiscence	4	1
Surgical site		
Haemorrhage	1	0.25
Cardiac		
MI	4	1
Arrhythmia	9	2.5
Vascular		
VTE	3	0.75
CVA	2	0.5
Urinary		
Renal failure	4	1
Acute urinary retention	3	0.75
Gastrointestinal		
Diarrhoea (noninfective)	4	1
Ileus	8	2.25
Complication severity		
Clavien-Dindo Grade		
0	239	63
1	36	10
2	72	20
3	18	4
4	8	2
5	4	1

*SSI* surgical site infection, *RSI* remote site infection, *UTI* urinary tract infection, *MI* myocardial infarction, *VTE* venous thromboembolism, *CVA* cerebrovascular accident

**Table 2:** Patient characteristics by severity of complication following surgery for colorectal cancer

Characteristic	All	Clavien-Dindo complication grade				
		0^a^	1–2^b^	3–4^c^	5^d^	*p*
*N* (%)	377 (100)	239 (63)	108 (30)	26 (7)	4 (1)	–
Age (<65/65–74/>74)	122/149/106	82/96/61	31/44/33	9/7/10	0/2/2	0.451
Sex (male/female)	208/169	116/123	74/34	15/11	3/1	0.005
BMI (<20/20–25/36–30/>30)	16/112/114/90	12/69/75/55	3/32/31/28/94	1/8/8/6	0/3/0/1	0.833
ASA (1/2/3/4)	48/169/145/14	36/112/83/7	9/45/48/6	3/11/12/0	0/1/2/1	0.014
Smoking (never/ex/current)	159/150/61	114/89/31	37/46/23	8/11/7	0/4/0	0.015
Preop mGPS (0/1/2)	284/37/56	180/23/36	82/9/17	18/5/3	4/0/0	0.636
Site (colon/rectum)	239/138	160/79	63/45	16/10	0/4	0.024
TNM stage (0/I/II/III)	10/80/159/128	8/54/101/76	1/20/43/44	0/5/14/7	1/1/1/1	0.120
Neoadjuvant treatment (no/yes)	299/65	191/40	84/19	21/5	3/1	0.970
Approach (open/laparoscopic)	266/110	162/77	83/24	18/8	3/1	0.323
Surgery > 4 h (yes/no)	83/247	51/154	22/76	9/16	1/1	0.456
Stoma (yes/no)	115/262	65/174	39/69	8/18	1/1	0.087
POD 2 CRP > 150 mg/L (yes/no)	205/162	114/117	72/35	16/9	3/1	0.004
POD 3 CRP > 150 mg/L (yes/no)	187/169	100/124	67/38	19/5	1/2	<0.001
POD 4 CRP > 150 mg/L (yes/no)	126/200	58/137	51/51	16/10	1/2	<0.001
Adjuvant treatment (no/yes)	269/108	171/68	73/35	21/5	4/0	0.323

*mGPS* preoperative modified Glasgow Prognostic score (0 = CRP < 10 mg/L, 1 = CRP ≥ 10 mg/L and albumin ≥ 35 g/L, 2 = CRP ≥ 10 mg/L and albumin < 35 g/L)

*POD* postoperative day

^a^0 = no complication

^b^1–2 = complication requiring minor intervention

^c^3–4 = complication requiring significant intervention

^d^5 = death

### Follow-Up

After exclusion of postoperative mortality (4, 1 %), death due to any cause occurred in 81 patients (22 %); 53 (14 %) were cancer-specific. The median follow-up for patients alive at the time of their censoring was 46 (range 24–86) months.

### Disease-Specific Survival

On univariate analysis (Table 3), age [hazard ratio (HR) 1.54, 95 % confidence interval (CI) 1.08–2.21, *p* = 0.018], ASA grade (HR 1.69, 95 % CI 1.16–2.46, *p* = 0.007), TNM stage (HR 2.50, 95 % CI 1.63–3.85, *p* < 0.001), mGPS (HR 1.67, 95 % CI 1.23–2.26, *p* = 0.001), breaching the established CRP threshold of 150 mg/L on POD 3 (HR 1.84, 95 % CI 1.01–3.35, *p* = 0.047) and POD 4 (HR 2.53, 95 % CI 1.43–4.48, *p* = 0.001) and complication severity (HR 1.66, 95 % CI 1.13–2.43, *p* = 0.009) were associated with disease-specific survival and included in multivariate analysis. On multivariate analysis (Table 3), ASA grade (HR 1.52, 95 % CI 1.01–2.28, *p* = 0.044), mGPS (HR 1.49, 95 % CI 1.08–2.07, *p* = 0.016), TNM stage (HR 2.46, 95 % CI 1.52–3.96, *p* < 0.001), and breaching the established CRP threshold of 150 mg/L on POD 4 (HR 2.00, 95 % CI 1.12–3.59, *p* = 0.020) remained independently associated with poorer disease-specific survival. Breaching the established CRP threshold of 150 mg/L on POD 3 was not included in multivariate analysis, because it was directly associated with breaching the established CRP thresholds of 150 mg/L on POD 4, which had a greater statistical significance on univariate analysis.

**Table 3:** Impact of the severity of postoperative complications on survival following surgery for colorectal cancer, univariate and multivariate survival analysis

Survival	Variable	Univariate HR (95 % CI)	*p*	Multivariate HR (95 % CI)	*p*
DSS	Age	1.54 (1.08–2.21)	0.018	–	0.225
	Sex	0.77 (0.45–1.32)	0.344	–	–
	BMI	0.88 (0.62–1.23)	0.446	–	–
	ASA	1.69 (1.16–2.46)	0.007	1.52 (1.01–2.28)	0.044
	Smoking	1.00 (0.69–1.46)	0.984	–	–
	mGPS	1.67 (1.23–2.26)	0.001	1.49 (1.08–2.07)	0.016
	Rectal	1.00 (0.57–1.74)	0.998	–	–
	TNM stage	2.50 (1.63–3.85)	<0.001	2.46 (1.52–3.96)	<0.001
	Neoadjuvant treatment	1.23 (0.63–2.39)	0.548	–	–
	POD 2 CRP > 150 mg/L	1.62 (0.91–2.89)	0.101	–	–
	POD 3 CRP > 150 mg/L	1.84 (1.01–3.35)	0.047	–	–
	POD 4 CRP > 150 mg/L	2.53 (1.43–4.48)	0.001	2.00 (1.17–3.59)	0.020
	Clavien-Dindo grade	1.66 (1.13–2.43)	0.009	1.51 (0.98–2.33)	0.061
	Adjuvant treatment	0.78 (0.42–1.46)	0.432	–	–
OS	Age	1.83 (1.36–2.48)	<0.001	1.35 (0.97–1.87)	0.074
	Sex	1.06 (0.68–1.64)	0.799	–	–
	BMI	0.85 (0.65–1.12)	0.242	–	–
	ASA	1.92 (1.41–2.61)	<0.001	1.49 (1.05–2.10)	0.024
	Smoking	1.20 (0.89–1.61)	0.238	–	–
	mGPS	1.52 (1.18–1.96)	0.001	–	0.170
	Rectal	0.78 (0.49–1.25)	0.308	–	–
	TNM stage	1.70 (1.25–2.31)	0.001	2.12 (1.45–3.41)	<0.001
	Neoadjuvant treatment	0.97 (0.54–1.73)	0.914	–	–
	POD 2 CRP > 150 mg/L	1.99 (1.22–3.26)	0.006	–	–
	POD 3 CRP > 150 mg/L	1.76 (1.08–2.85)	0.022	–	–
	POD 4 CRP > 150 mg/L	2.02 (1.27–3.20)	0.003	2.14 (1.34–3.41)	0.001
	Clavien-Dindo grade	1.30 (0.93–1.81)	0.127	–	–
	Adjuvant treatment	0.64 (0.37–1.09)	0.098	0.33 (0.17–0.64)	0.001

*HR* hazard ratio, *CI* confidence interval, *DSS* disease-specific survival, *OS* overall survival, *mGPS* modified Glasgow Prognostic score, *POD* postoperative day

### Overall Survival

On univariate analysis (Table 3), age (HR 1.83, 95 % CI 1.36–2.48, *p* < 0.001), ASA grade (HR 1.92, 95 % CI 1.41–2.61, *p* < 0.001), mGPS (HR 1.52, 95 % CI 1.18–1.96, *p* = 0.001), TNM stage (HR 1.70, 95 % CI 1.25–2.31, *p* = 0.001), breaching the established CRP threshold of 150 mg/L on POD 2 (HR 1.99, 95 % CI 1.22–3.26, *p* = 0.006, POD 3 (HR 1.76, 95 % CI 1.08–2.85, *p* = 0.022), and POD 4 (HR 2.02, 95 % CI 1.27–3.20, *p* = 0.003), and adjuvant treatment (HR 0.64, 95 % CI 0.37–1.09, *p* = 0.098) were associated with overall survival and included in multivariate analysis. On multivariate analysis (Table 3), ASA grade (HR 1.49, 95 % CI 1.05–2.10, *p* = 0.024), TNM stage (HR 2.12, 95 % CI 1.45–3.09, *p* < 0.001), breaching the established CRP threshold of 150 mg/L on POD 4 (HR 2.14, 95 % CI 1.34–3.41, *p* = 0.001), and adjuvant treatment (HR 0.33, 95 % CI 0.17–0.64, *p* = 0.001) all remained independently associated with overall survival. Breaching the established CRP threshold of 150 mg/L on POD 2 and 3 were not included in multivariate analysis, because they were directly associated with breaching the established CRP thresholds of 150 mg/L on POD 4, which had a greater statistical significance on univariate analysis.

### Colonic Resection

When the subgroup of 239 patients who underwent surgery for colonic cancer were considered, 79 (33 %) experienced complications (Table 4). No patients died within 30 days of surgery or during the same admission. When classified using the Clavien-Dindo scale, 63 were grade 1–2 and 16 were grade 3–4. When patient’s demographic, pathological, and clinical characteristics were compared across complication severity (Table 3), only smoking status (*p* = 0.047) was significantly associated. There was a significant association between complication severity and the proportion of patients breaching the established CRP threshold of 150 mg/L on POD 2 (*p* = 0.032), POD 3 (*p* = 0.002), and POD 4 (*p* = 0.005).

**Table 4:** Patient characteristics by severity of complication following surgery for colonic cancer

Characteristic	All	Clavien-Dindo complication grade				
		0^a^	1–2^b^	3–4^c^	5^d^	*p*
*N* (%)	239	160	63	16	0	–
Age (<65/65–74/>74)	66/88/85	48/59/53	14/24/25	4/5/7	0/0/0	0.724
Sex (male/female)	127/112	77/83	42/21	8/8	0/0/0	0.111
BMI (<20/20–25/36–30/>30)	11/64/68/60	9/41/50/38	1/19/12/19	1/4/6/3	0/0/0/0	0.430
ASA (1/2/3/4)	24/99/105/11	20/70/63/7	2/23/34/4	2/6/8/0	0/0/0/0	0.227
Smoking (never/ex/current)	103/91/40	79/54/23	20/30/12	4/7/5	0/0/0	0.047
Preop mGPS (0/1/2)	170/28/41	115/18/27	46/6/11	9/4/3	0/0/0	0.513
TNM stage (0/I/II/III)	0/50/112/77	0/33/73/54	0/14/29/20	0/3/10/3	0/0/0/0	0.734
Approach (open/laparoscopic)	83/156	57/103	20/43	6/10	0/0	0.836
Surgery > 4 h (yes/no)	28/179	17/118	7/50	4/11	0/0	0.303
Stoma (yes/no)	14/158	7/113	6/37	1/8	0/0	0.277
POD 2 CRP > 150 mg/L (yes/no)	129/105	78/78	43/19	8/8	0/0	0.032
POD 3 CRP > 150 mg/L (yes/no)	121/105	69/81	41/20	11/4	0/0	0.002
POD 4 CRP > 150 mg/L (yes/no)	81/117	41/84	31/26	9/7	0/0	0.005
Adjuvant treatment (no/yes)	69/170	48/112	18/45	3/13	0/0	0.638

*mGPS* preoperative modified Glasgow Prognostic score (0 = CRP < 10 mg/L, 1 = CRP ≥ 10 mg/L and albumin ≥ 35 g/L, 2 = CRP ≥ 10 mg/L and albumin < 35 g/L)

*POD* postoperative day

^a^0 = no complication

^b^1–2 = complication requiring minor intervention

^c^3–4 = complication requiring significant intervention

^d^5 = death

On multivariate analysis (Table 5) mGPS (HR 1.81, 95 % CI 1.20–2.72, *p* = 0.005), TNM stage (HR 2.28, 95 % CI 1.23–4.21, *p* = 0.009), and breaching the established CRP threshold of 150 mg/L on POD 4 (HR 2.42, 95 % CI 1.13–5.18, *p* = 0.023) were independently associated with disease specific survival after surgery for colonic cancer. ASA grade (HR 1.99, 95 % CI 1.28–3.10, *p* = 0.002), mGPS (HR 1.53, 95 % CI 1.11–2.10, *p* = 0.010), and breaching the established CRP threshold of 150 mg/L on POD 4 (HR 2.32, 95 % CI 1.29–4.20, *p* = 0.005) were independently associated with overall survival after surgery for colonic cancer.

**Table 5:** Impact of the severity of postoperative complications on survival following surgery for colonic cancer, univariate and multivariate survival analysis

Survival	Variable	Univariate HR (95 % CI)	*p*	Multivariate HR (95 % CI)	*p*
DSS	Age	1.52 (0.96–2.41)	0.073	–	0.316
	Sex	0.72 (0.37–1.44)	0.356	–	–
	BMI	0.69 (0.44–1.09)	0.109	–	–
	ASA	1.63 (1.00–2.67)	0.051	1.70 (0.99–2.93)	0.057
	Smoking	0.94 (0.58–1.52)	0.792	–	–
	mGPS	1.95 (1.34–2.82)	<0.001	1.81 (1.20–2.72)	0.005
	TNM stage	2.27 (1.32–3.90)	0.003	2.28 (1.23–4.21)	0.009
	POD 2 CRP > 150 mg/L	1.69 (0.82–3.48)	0.157	–	–
	POD 3 CRP > 150 mg/L	1.97 (0.89–4.36)	0.094	–	–
	POD 4 CRP > 150 mg/L	2.78 (1.31–5.91)	0.008	2.42 (1.13–5.18)	0.023
	Clavien-Dindo grade	1.34 (1.01–1.78)	0.043	–	0.164
	Adjuvant treatment	0.77 (0.35–1.70)	0.516	–	–
OS	Age	1.78 (1.23–2.57)	0.002	1.43 (0.94–2.15)	0.092
	Sex	1.04 (0.61–1.78)	0.873	–	–
	BMI	0.75 (0.53–1.06)	0.100	–	–
	ASA	1.98 (1.34–2.93)	0.001	1.99 (1.28–3.10)	0.002
	Smoking	1.19 (0.83–1.70)	0.354	–	–
	TNM stage	1.53 (1.04–2.25)	0.030	–	0.114
	mGPS	1.66 (1.23–2.23)	0.001	1.53 (1.11–2.10)	0.010
	POD 2 CRP > 150 mg/L	2.18 (1.20–3.96)	0.010	–	–
	POD 3 CRP > 150 mg/L	1.88 (1.03–3.42)	0.040	–	–
	POD 4 CRP > 150 mg/L	2.33 (1.31–4.17)	0.004	2.32 (1.29–4.20)	0.005
	Clavien-Dindo grade	1.08 (0.84–1.39)	0.548	–	–
	Adjuvant treatment	0.67 (0.35–1.27)	0.205	–	–

*HR* hazard ratio, *CI* confidence interval, *DSS* disease-specific survival, *OS* overall survival, *mGPS* modified Glasgow Prognostic score, *POD* postoperative day

## Discussion

The results of the present study report a significant association between the magnitude of the postoperative systemic inflammatory response and complication severity, using the Clavien-Dindo grade, following surgery for colorectal cancer. Furthermore, the magnitude of the postoperative systemic inflammatory response, in particular CRP on POD 4, was significantly associated with disease-specific and overall survival independent of postoperative complications. These relationships remained in a subgroup of patients who underwent colonic surgery. Therefore, the present results would suggest that the relationship between postoperative complication severity and poorer long-term survival is, at least in part, dependent on the magnitude of the postoperative systemic inflammatory response.

The results of the present study are consistent with previous studies showing an association between male gender, preoperative ASA grade, smoking status, and complication severity following colorectal surgery.^19^,^25^,^26^ Moreover, with two recent studies reporting the association between complication severity and the magnitude of the postoperative systemic inflammatory response in patients with colorectal cancer and also in patients undergoing surgery for gastric and oesophageal cancer.^20^–^23^

A recent meta-analysis reported that complication type and severity were independently associated with poorer oncologic outcomes following colorectal surgery and liver resection for colorectal cancer.^15^ However, the present study is the first to include a measure of the magnitude of the systemic inflammatory response together with the severity of complication in survival analysis following surgery for colorectal cancer. Although the relationship between postoperative infective complications and poorer survival in patients with colorectal cancer has been extensively documented, complication severity using the Clavien-Dindo scale provides a validated, objective framework for the definition of such postoperative complications.^10^

Taken together the implications of these results are important. They would suggest that the mechanisms that link the magnitude of the postoperative systemic inflammatory response, postoperative complications, and poorer oncological outcomes are inflammatory in aetiology.^27^ In previous work, it has been reported that the presence of preoperative systemic inflammation, as measured by the mGPS, but not postoperative complication, was associated with poorer long-term outcomes following surgery for colorectal cancer.^8^ However, the magnitude of the postoperative systemic inflammatory response was not considered. More recently, it is now recognised that the magnitude of the systemic inflammatory response following surgery for colorectal cancer is associated with the extent of postoperative complications.^17^,^18^,^20^,^21^ The present study shows that both the pre- and postoperative systemic inflammatory responses are associated with oncologic outcomes independent of tumour stage and postoperative complications.

The exact mechanisms underlying these relationships are unclear. However, the presence of an innate immune driven systemic inflammatory response can suppress cytotoxic immunity and may promote the development of postoperative complications and tumour progression.^28^–^30^ If this were proven to be the case, it therefore would be rational to consider the postoperative systemic inflammatory response a target for therapeutic intervention. Clearly, such therapeutic intervention also would test the above hypothesis, because it would be anticipated that a reduction in the postoperative systemic inflammatory response would not only result in a reduction in the severity of postoperative complications but also improve long-term outcomes, not only in colorectal cancer surgery, but in surgery for all solid tumours. It remains to be determined whether the modulation of the postoperative systemic inflammatory response may reduce the frequency and/or severity of postoperative complications or improve long-term outcomes following surgery for colorectal cancer.

Therefore, it is of interest that several meta-analyses investigating trials of preoperative corticosteroids in major abdominal and gastrointestinal surgery have suggested a reduction in both the magnitude of the postoperative systemic inflammatory response and postoperative complications.^31^–^33^ The long-term outcomes of these trials however is less clear; two, small follow-up studies suggested that preoperative corticosteroids had a potentially negative oncologic impact.^34^,^35^ However, preoperative corticosteroids have been widely incorporated into perioperative care guidelines for colorectal surgery due to their efficacy in the reduction of rates of postoperative nausea and vomiting.^36^ Other strategies might include the use of NSAIDs, H_2_ receptor antagonists, or statins in the perioperative period, each of which have been suggested to attenuate the systemic inflammatory response in patients with cancer.^29^,^37^ Additionally, laparoscopic surgery has been associated with a reduction in the magnitude of the postoperative systemic inflammatory response where technically appropriate.^38^

A main limitation of the present study was the relatively short follow-up period. In addition, a relatively small number of Clavien-Dindo grade 3–4 complications occurred. The significant difference in frequency of severe complication between colonic and rectal resection led to the separate analysis of patients undergoing colonic resection. Nevertheless, comparative analysis showed similar significant relationships with survival compared with the whole cohort.

In summary, the results of the present study show that the magnitude of the postoperative systemic inflammatory response was associated with oncologic outcome following surgery for colorectal cancer, independent of postoperative complications, or disease stage.
